# Evaluation on the Mechanical Properties of Ground Granulated Blast Slag (GGBS) and Fly Ash Stabilized Soil via Geopolymer Process

**DOI:** 10.3390/ma14112833

**Published:** 2021-05-26

**Authors:** Syafiadi Rizki Abdila, Mohd Mustafa Al Bakri Abdullah, Romisuhani Ahmad, Shayfull Zamree Abd Rahim, Małgorzata Rychta, Izabela Wnuk, Marcin Nabiałek, Krzysztof Muskalski, Muhammad Faheem Mohd Tahir, Syafwandi, Muhammad Isradi, Marek Gucwa

**Affiliations:** 1 Centre of Excellence Geopolymer and Green Technology (CEGeoGTech), Universiti Malaysia Perlis, Kangar 01000, Perlis, Malaysia; romisuhani@unimap.edu.my (R.A.); shayfull@unimap.edu.my (S.Z.A.R.); faheem@unimap.edu.my (M.F.M.T.); 2 Faculty of Chemical Engineering Technology, Universiti Malaysia Perlis (UniMAP), Kangar 01000, Perlis, Malaysia; 3 Faculty of Mechanical Engineering Technology, Universiti Malaysia Perlis, Arau 02600, Perlis, Malaysia; 4 Department of Physics, Częstochowa University of Technology, Generała Jana Henryka Dąbrowskiego 69, 42-201 Częstochowa, Poland; malgorzata.rychta@pcz.pl (M.R.); wnuk.izabela@wip.pcz.pl (I.W.); nmarcell@wp.pl (M.N.); krzysztof.muskalski@pcz.pl (K.M.); 5 Faculty of Civil Engineering, Mercu Buana Universiti, Jakarta 11650, Indonesia; syafwandi@mercubuana.ac.id (S.); isradi@mercubuana.ac.id (M.I.); 6 Faculty of Mechanical Engineering and Computer Science, Częstochowa University of Technology, Generała Jana Henryka Dąbrowskiego 69, 42-201 Częstochowa, Poland; mgucwa@spaw.pcz.pl

**Keywords:** geopolymer, clay soil, fly ash, ground granulated blast slag, soil stabilization

## Abstract

This study intended to address the problem of damaged (collapsed, cracked and decreased soil strength) road pavement structure built on clay soil due to clay soil properties such as low shear strength, high soil compressibility, low soil permeability, low soil strength, and high soil plasticity. Previous research reported that ground granulated blast slag (GGBS) and fly ash can be used for clay soil stabilizations, but the results of past research indicate that the road pavement construction standards remained unfulfilled, especially in terms of clay’s subgrade soil. Due to this reason, this study is carried out to further investigate soil stabilization using GGBS and fly ash-based geopolymer processes. This study investigates the effects of GGBS and ratios of fly ash (solid) to alkaline activator (liquid) of 1:1, 1.5:1, 2:1, 2.5:1, and 3:1, cured for 1 and 7 days. The molarity of sodium hydroxide (NaOH) and the ratio of sodium silicate (Na_2_SiO_3_) to sodium hydroxide (NaOH) was fixed at 10 molar and 2.0 weight ratio. The mechanical properties of the soil stabilization based geopolymer process were tested using an unconfined compression test, while the characterization of soil stabilization was investigated using the plastic limit test, liquid limit test, scanning electron microscopy (SEM), X-ray diffraction (XRD), and Fourier transform infrared spectroscopy (FTIR). The results showed that the highest strength obtained was 3.15 MPA with a GGBS to alkaline activator ratio of 1.5 and Na_2_SiO_3_ to NaOH ratio of 2.0 at 7 days curing time. These findings are useful in enhancing knowledge in the field of soil stabilization-based geopolymer, especially for applications in pavement construction. In addition, it can be used as a reference for academicians, civil engineers, and geotechnical engineers.

## 1. Introduction

Soil is a mixture of rock or mineral particles, water, and air. Based on these constituents, the soil properties may vary from one region to another. In addition, different soil types behave differently for the purposes of building work [1,2]. In construction engineering, soil layers are often found to have a low bearing capacity, which significantly affects the various stages of construction involved, such as planning, implementation, operational, and maintenance stages [3,4]. In fact, there are multiple types of soil with different characteristics which depend on the location. This research focuses explicitly on the clay soil on which road pavements are built [3,5]. Clay soil, with its low strength and stiffness, may cause damage to the foundation of buildings and cracks along the road pavement. Thus, it poses a challenge for experts and companies engaged in civil engineering [5,6]. In addition, clay soil is considered a potential hazard that inflicts severe damage to the engineering structure [6,7].

Furthermore, structures built on clay soils are subjected to extensive damages due to the adverse and unpredictable characteristics of the soil [4,5,6,7,8]. Usually, the soil will shrink with the reduction of moisture content and swell with the increase in moisture content [8,9]. Clay soil has high plasticity, low support, and high shrinkage when the soil contains water [9,10]. This condition encouraged civil engineers to seek solutions in order to strengthen the soil through soil stabilization [3,4,5,6,7,8,9,10,11,12,13]. Soil stabilization aims to increase the mechanical properties of the soil and also to control plasticity and shrinkage in soils containing excess water [4,8,9,10]. In order to successfully withstand superstructure loads, soil stabilization techniques are essential to ascertain the excellent stability of soil [6,9,10].

The soil improvement technique by adding ground granulated blast slag (GGBS) or fly ash has been used in geotechnical engineering works [13,14]. Examples of these works are in building foundations, highways, dams, canals, and other embankment works [7,11,12]. Previous research has reported that adding GGBS or fly ash can improve the strength characteristics of soils [13,14,15,16,17]. Sharman et al. [18] investigated the performance of fly ash and GGBS for soil stabilization applications. The effects of adding GGBS and fly ash were observed at 7, 14, and 28 days of curing. It was reported that the value of the plasticity index and liquid limit on the clay soil decreased, and the highest soil strength was obtained at 28 days curing time with a strength value of 0.45 MPa. The results showed that combining GGBS and fly ash to form a binder provides a new opportunity to increase pozzolan activity, which can increase the unconfined compression and reduce the swelling potential of clay soil [14,15,16,17,18]. However, the properties obtained still do not comply with the soil stabilization standard for road construction application based on ASTM D 4609 [19], especially in terms of the pre-determined subgrade layers [19,20,21]. The unconfined compressive strength of soil should be more than 0.8 MPa at 7 days of curing time [21]. In addition, the research conducted by Sharman et al. [18] did not consider controlling the swelling potential, which is essential in maintaining the plastic and liquid limits in the clay soil. This is important to avoid building or road structures from collapsing. Anil Kumar et al. [18] used water without an alkali activator for a soil stabilization mixing process where the optimum strength achieved was only 0.45 MPa within 28 days, which still does not comply with the minimum compressive strength required for subgrade layers based on ASTM D 4609 [19,20,21].

Therefore, it is necessary to carry out further studies on the potential of adding GGBS and fly ash as soil stabilizers with the aim of increasing the soil compression strength, controlling the swelling potential of clay soil, and reducing the curing time to achieve a maximum power of soil [22,23]. The civil engineering industry has always been keen on searching for an environmentally friendly, alternative solution with low carbon dioxide emission as a new and sustainable material to replace Portland cement as a soil stabilizer. Recently, the use of geopolymers as a green material has shown to be an excellent alternative to Portland cement for enhancing weak soil [22,23,24]. Geopolymers have impressive engineering properties, including greater strength and better adhesion to soil properties [22,23,24,25,26]. Furthermore, geopolymer is the synthesis of inorganic natural materials through the polymerization process [26,27,28]. The primary basic materials needed for the manufacturing of this geopolymer material are materials that consist of high alumina (Al) and silica (Si) elements. The elements of Si and Al include industrial by-products, such as fly ash from coal combustion and GGBS from iron combustion for geopolymer processes in soil stabilization [22,23,24,25,26,27,28,29,30]. In order to dissolve the elements of Si and Al and also to allow chemical reactions to occur, alkaline solutions such as sodium silicate (Na_2_SiO_3_) and sodium hydroxide (NaOH) are used [22,23,24,25,26,27,28,29,30,31]. Previous research on soil stabilization based geopolymer using waste materials such as GGBS and fly ash did not focus on the road construction application, especially in the subgrade layer [14,15,16,17,18,22,23,24,25].

This research is an initiative to realize the capability of the soil stabilization-based geopolymer to be used as a subgrade layer. To achieve the objective of this research, unconfined compression test, plastic limit test, liquid limit test, scanning electron microscopy (SEM), X-ray diffraction (XRD), and Fourier transform infrared spectroscopy (FTIR) were carried out to determine the performance of soil stabilization based geopolymer using GGBS and fly ash. The result of this research will be an academic reference for the development of soil stabilization based on geopolymer science that uses GGBS and fly ash in road construction, especially in the subgrade layers of the soil.

## 2. Materials and Experimental Method

### 2.1. Sources of Raw Materials

The raw materials used in this study were soil, fly ash, and ground granulated blast slag (GGBS), which were used as the primary alumino-silicate source materials. In this research, soil samples were taken at Kok Klang, Perlis, Malaysia. This location was chosen because it has a clay soil type, thus having the potential for high shrinkage, which may lead to collapse or damage of road construction. Fly ash samples used in this research were collected from a coal combustion plant located at Manjung Power Station, Perak, Malaysia. The samples of GGBS were obtained from YTL Cement Marketing Sdn. Bhd., Kuala Lumpur, Malaysia. Chemical reagents such as sodium silicate (Na_2_SiO_3_) with a composition of 30.1% silica oxide (SiO_2_) were supplied by South Pacific Chemical Industries Sdn. Bhd (SPCI), Malaysia, and sodium hydroxide (NaOH) micro-pearls with 99.0% purity were provided by Formosa Plastic Corporation Sdn. Bhd.

### 2.2. Characterization of Raw Materials

Characterization of raw materials techniques such as particle size distribution test, specific gravity test, Atterberg limit test, compaction test, Unified Soil Classification System (USCS), and chemical analysis were considered. The particle size distribution test of the clay soil was measured in accordance with the ASTM D-422 [32]. The specific gravity was determined using the ASTM D-854 [33]. The Atterberg limit was measured in accordance with the BS 1377-2 [34]. The compaction test of the soil was measured in accordance with the ASTM D-698 [35]. The soil type was determined based on a unified soil classification system (USCS) using the data obtained from the particle size distribution, specific gravity, and Atterberg limit test. Chemical analyses of soil, fly ash, and ground granulated blast slag was performed using an X-ray fluorescence (XRF) spectrometer (PANalytical PW4030, MiniPAL 4, Malvern Panalytical, Worcestershire, UK), X-ray diffractometer (XRD-6000, Shimadzu, Columbia, MD, USA), Fourier transform infrared using FTIR spectrometer (RX1 Perkin Elmer, Llantrisant, UK) spectroscopy, and scanning electron microscope (JSM-6460LA, JEOL, Tokyo, Japan). All testing was performed at laboratories in the Faculty of Chemical Engineering and Technology, Universiti Malaysia Perlis, Perlis, Malaysia.

### 2.3. Method

The alkaline activator used in this research is a blend of sodium hydroxide (NaOH) and sodium silicate (Na_2_SiO_3_). This is because NaOH contributes to the reaction of the elements Al and Si contained in GGBS and fly ash, thus producing a solid polymer bond. On the other hand, Na_2_SiO_3_ contributes as a catalyst that accelerates chemical reactions [22,23,24,25,26,27,28,29,30,31]. The concentration of NaOH used was 10 M, and it was used for the manufacture of alkali activator by mixing the NaOH and Na_2_SiO_3_ solutions in a ratio of 2.0 weight, respectively. This can be referred to in the study reported by Abdullah et al. [31], which stated that the optimum compression strength could be achieved by using a 10 M concentration of NaOH with a solution ratio of 2.0 (NaOH to Na_2_SiO_3_). The alkali activator can be produced by mixing NaOH and Na_2_SiO_3_ solutions, and it can be used after 24 h due to the occurrence of polymerization that releases a massive amount of heat [26,31,36].

In the mixing process, raw materials (GGBS, fly ash, and soil) were dried and mixed together. All specimens, as tabulated in Table 1, are produced using various ratios of GGBS or fly ash to alkali activator and Na_2_SiO_3_/NaOH as reported by Phummiphan and Shamshad [14,15]. Then, the samples were cured at ambient temperature for 1 to 7 days. This is due to the optimum compression test, which cannot be obtained if the curing time is less than 1 day [30]. In addition, the molarity of NaOH and ratio of NaOH to Na_2_SiO_3_ were fixed at 10 M and 2.0, respectively [31]. The unconfined compression test was conducted according to ASTM D 2166 standard [37] in order to evaluate the mechanical strength of every clay soil sample. The mixed designs are presented in Table 1.

Afterwards, soil and GGBS or fly ash were blended to yield an even GGBS or fly ash content. Then, the alkali activator is spilt into the blended powder until the mixture becomes homogeneous. Next, the mixture is poured into the pre-oiled cylindrical mold with 38 mm diameter and 76 mm height and compacted until solid for unconfined compression test (UCT) sample. The samples were removed from the mold with an extruder and sealed by using plastic to prevent moisture loss. All samples for UCT were cured for 1 and 7 days to obtain the optimum strength [26,31]. Through the above procedure, the UCT value is obtained, which has an impact on strengthening the subgrade layer of road construction.

#### 2.3.1. Unconfined Compression Test

Unconfined compression test (UCT) was performed using motorized unconfined compression (NL 5023 X, NL Scientific Instruments Sdn Bhd, Selangor, Malaysia) according to the ASTM D 2166 [37] standard. Unconfined compressive strength will be obtained as the optimum load achieve per unit area at 15% axial strain [38].

#### 2.3.2. Liquid Limit

The liquid limit of clay soil was measured following the BS 1377-2 [34] standard procedure using a digital cone penetrometer (NL 5003 X/002, NL Scientific Instruments Sdn Bhd, Selangor, Malaysia). The reading of the penetration cone was documented for each clay soil sample. The initial penetration reading must be at 15 mm depth; if not, the water must be added to achieve the required depth. The water content for each clay soil sample was determined by taking 20 g of clay soil samples from the region penetrated by the digital cone penetrometer. After that, each sample of clay soil was tested four times with the addition of water. When the groove closes for 1/2 inch after 25 drops of the cup, the moisture content was defined as the liquid limit.

#### 2.3.3. Plastic Limit

The plastic limit of clay soil was measured following the BS 1377-2 [34] standard. The samples of clay soil were sieved using 425 µm sieving mesh and then mixed with water. The plastic limit is determined by repeatedly remolding a small ball of moist plastic soil and manually rolling it out into a 1/8 inch (3.2 mm) thread. Then, the soil samples were divided into three sub-samples of soil weighing 10 g for each sub-sample of soil. The subsamples of soil were rolled up by hand and molded manually to the sub-sample size of 3 mm diameter. The plastic limit is the moisture content at which the thread crumbles before being completely rolled out. Finally, the samples were placed into the oven for 24 h to determine the water content.

#### 2.3.4. X-ray Diffraction (XRD)

The phase analysis of soil-based geopolymer samples was analyzed using X-ray diffractometer (XRD-6000, Shimadzu, Columbia, MD, USA), with Cu-Kα radiation composed at 35 mA and 40 kV, at scan range of 10° to 80° and at a step size of 0.02°, integrated at a rate of 1.0 s per step.

#### 2.3.5. Scanning Electron Microscope (SEM)

The morphology analysis of clay soil-based geopolymer sample was conducted using scanning electron microscope (JSM-6460LA, JEOL, Tokyo, Japan). The clay soil-based geopolymer sample was coated with a thin layer of gold using a sputter coater to become conductive. The coated sample was mounted using carbon tape and placed in the instrument for analysis.

#### 2.3.6. Fourier Transformation Infrared Spectroscopy (FTIR)

The FTIR was performed to analyze the functional group of clay soil-based geopolymer samples. The soil samples were evaluated using an FTIR spectrometer (RX1 Perkin Elmer, Llantrisant, UK) with a scan ranging from 600 until 4000 cm^−1^ with a scan time of 5 min.

## 3. Results and Discussion

### 3.1. Raw Materials Characterization

#### 3.1.1. Physical Properties

The general properties of the soil, fly ash, and GGBS used in this study are tabulated in Table 2. Based on the results from the particle distribution analysis performed on the soil, fly ash and GGBS indicated that the soil has varying particle sizes since it comprises 48%, 6%, 4%, and 52%, 94%, 96% courses, and fine particles respectively. The specific gravity value of the soil was 2.686. From the clay soil compaction test, the optimum water content and the optimum dry density obtained were 15% and 1.66 g/cm^3^, respectively. According to ASTM D2487 [39], soil with the percentage of fine aggregates surpassing 50% is classified as fine-particle inorganic clay and high plasticity (CH). The fine particle of the fly ash and GGBS contributes by filling the void area between soil particles. This can cause clay soil to become stable and increase the clay soil’s compressive strength [15]. The liquid limit of soil, fly ash, and GGBS was recorded at 51.20%, 23.40%, and 40.73%, respectively. The percentage of soil recorded was 28.48% for the plasticity index, while no plastic limit value was recorded for fly ash and GGBS. The term non-plastic refers to the material having low cohesive value. Materials with low cohesive value have a lower plasticity index and controlled swelling behavior for clay soil.

#### 3.1.2. Chemical Properties

The percentage of chemical elements of the soil, fly ash, and GGBS used in this study are tabulated in Table 3. Based on the result, clay soil particles’ chemical elements consist of a rich amount of silica oxide (SiO_2_) and alumina oxide (Al_2_O_3_), where more than 90% was found for both elements. In order to allow the geopolymerization process to occur, the primary requirement had to be fulfilled where the materials used must be rich in silica (Si) and alumina (Al) minerals [26,27]. The fly ash used is composed primarily of SiO_2_ and Al_2_O_3_ minerals with a percentage of 30.70% and 13.30%, respectively, making fly ash appropriate to be used as a precursor for geopolymer [22]. The fly ash used in this research can be classified into Class C based on its chemical content, which consists of total silicon, aluminum, and iron less than 70%, according to ASTM C 618 [40]. The GGBS has a high calcium oxide content (CaO) of 50.37%. The total composition of silicon dioxide and aluminum oxide is 40.9%. The calcium oxide (CaO) content in GGBS will also increase the early strength of geopolymer produced [41,42,43,44]. GGBS and alkali activator solution reaction forms calcite (CaCO_3_) and calcium silicate hydrate (C-S-H) within the geopolymer matrix [45,46]. These hydration products contribute to higher strength along with the aluminosilicate structure in the GGBS samples [47,48,49,50].

**Figure 1:**
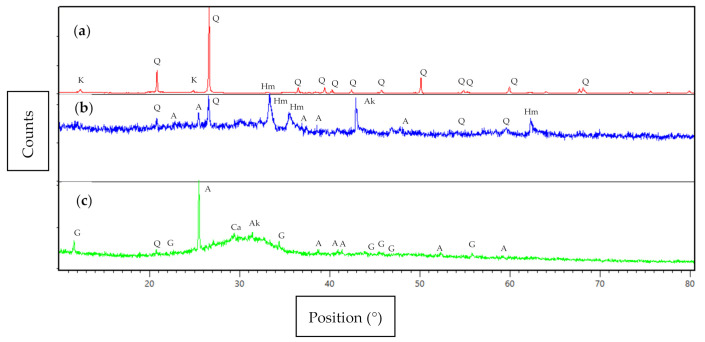
XRD patterns of (**a**) soil, (**b**) fly ash and (**c**) GGBS. Q: quartz (ICDD reference: 00-046-1045); K: kaoline (ICDD reference: 00-029-1488); Hm: hematite (ICDD reference: 00-024-0072); G: gypsum (ICDD reference: 00-037-1496); A: anhydrite (ICDD reference: 00-037-1496, 01-086-2270); Ak: akermanite (ICDD reference: 00-035-0592); Ca: calcite (ICDD reference: 01-089-0387).

Figure 1 shows the X-ray diffractometer of the soil, fly ash, and GGBS. The mineralogical component of soil is quartz (SiO_2_), hematite (Fe_2_O_3_), and kaolinite Al_2_Si_2_O_5_(OH)_4_. Kaolinite appears in clay soil as the plasticity index, and liquid limit were high due to clay minerals. The mineralogical components of fly ash consist of quartz (SiO_2_), hematite (Fe_2_O_3_), anhydrite (CaSO_4_), and akermanite (2CaO·MgO·2SiO_2_). Akermanite (2CaO·MgO·2SiO_2_) and anhydrite (CaSO_4_) in fly ash contribute to maintaining the volume expansion of soil. Hematite in fly ash strengthens the chemical bond during the geopolymerization process, leading to increased compressive strength [47]. The mineralogical component of GGBS consists of anhydrite (CaSO_4_), gypsum (CaSO_4_·2H_2_O), quartz (SiO_2_), calcite (CaCO_3_), and akermanite (2CaO·MgO·2SiO_2_). The presence of the quartz (SiO_2_), anhydrite (CaSO_4_), gypsum (CaSO_4_·2H_2_O), calcite (CaCO_3_), and akermanite (2CaO·MgO·2SiO_2_) with high intensity are due to the source of GGBS, which originates from high calcium (Ca), silica (Si) and low magnesium (Mg) content. The presence of mineral gypsum affects increasing the strength of soil [31]. The presence of quartz minerals in the soil, fly ash, and GGBS can contribute a reasonable amount of silicon to forming the Si–O–Si bond in the geopolymer, thus leading to higher compressive strength [48,49].

**Figure 2:**
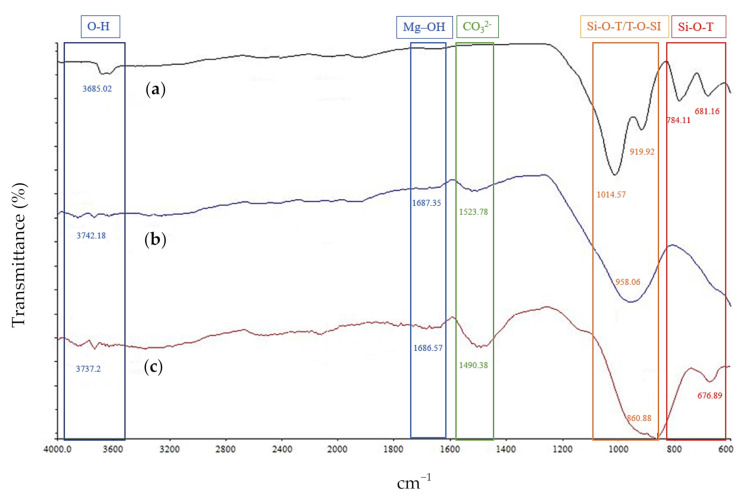
FTIR spectra of (**a**) soil, (**b**) fly ash, and (**c**) GGBS.

Figure 2 shows the presence of alumina (Al) and silica (Si) structure bond of soil, ground granulated blast slag (GGBS), and fly ash which was obtained using FTIR analysis. The existence of an aluminosilicate functional group was indicated by a wavenumber ranging between 800 cm^−1^ and 1100 cm^−1^. The asymmetric T-O-Si/Si-O-T stretching (T showing either Si or Al) of soil, GGBS, and fly ash were indicated at wavenumbers 1014.57, 958.06 and 860.88 cm^−1,^ respectively. Furthermore, the spectra peak in the range of 676.89 to 784.11 cm^−1^ is the bending vibration of Si–O–T bonds. The peaks in the range between 3685.02 and 3737.20 cm^−1^ are attributed to the O–H stretching vibration, which implies moisture in the raw materials. The band around 1600 cm^−1^ is attributed to the Mg–O bonds, indicating the magnesium mineral in the raw materials of fly ash and GGBS. This finding is supported by analyzing the chemical elements in Table 3, which indicates the existence of MgO (3.2% and 3.6%) in the fly ash and GGBS. The band 1490.38 cm^−1^ identified in the fly ash and GGBS is assigned to symmetric stretching of O–C–O bonds of carbonate group exposed to superficial weathering of fly ash and GGBS during storage. Besides, the band at circumference 1490.38 cm^−1^ is the characteristic of CO_3_ stretching, indicating the existence of calcite due to the reaction between excess calcium oxide and atmospheric carbon dioxide [50,51].

**Figure 3:**
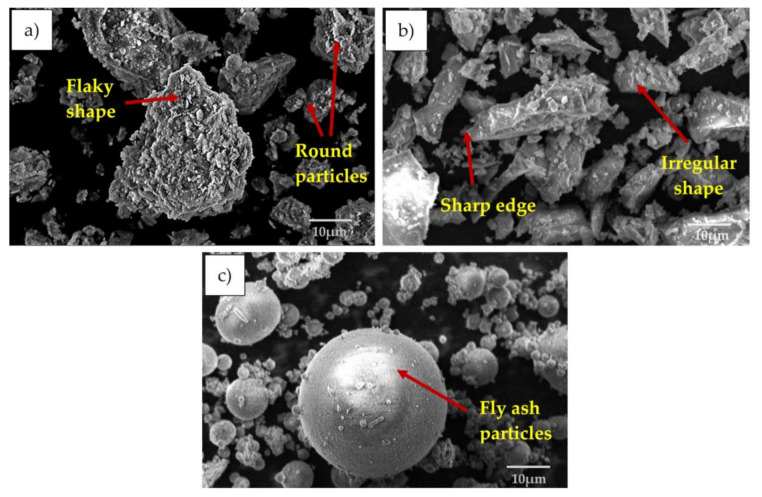
SEM image of (**a**) clay soil, (**b**) GGBS, and (**c**) fly ash.

The morphology of the soil, fly ash, and ground granulated blast slag (GGBS) were analyzed using scanning electron, as seen in Figure 3. Based on the result, the microstructure of soil consists of several flaky-like particles. Flaky particles of clay soil support a larger surface area. The large surface area allows the soil to hold more water quantity, which makes the high values of liquid limit and plasticity index of the clay soil clearer. Besides, the existence of few rounded particles in the microstructure indicates the presence of large voids between their particles as a result of the inexistence of cementing compound that could bind the soil particles together, taking over the low value of strength. The geopolymerization reaction to soil can also combine the particles of soil hence, induce the change of the morphology from having large voids to a dense appearance [26,31,47,50]. The microstructure of GGBS particles consists several of irregular-shaped and sharped-edged with a rough surface.

Furthermore, the shape of GGBS particles was affected by the processing approaches such as air mill, ball mill, and vibro mill. While GGBS is active with alkali activators due to the high hydration reaction rate, they form a desirable early strength acceptable for construction application [47,50,51]. The fly ash’s microstructure consists of a series of spherical vitreous particles of different sizes but with a standard smooth texture. Another study reported the geopolymerization reaction of the fly ash to the soil, making the distinct soil particles appear to have a more bound and dense texture in the stabilized material with the void professedly filled [18,22,23,27,47].

### 3.2. Unconfined Compression Test

**Figure 4:**
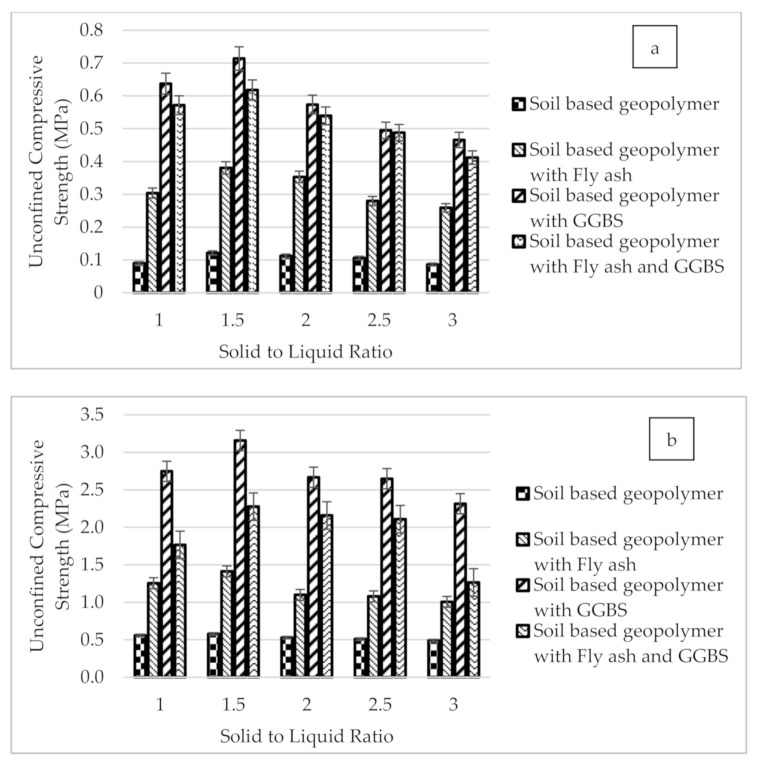
The effect of different solid to liquid (S/L) ratios of soil based geopolymer, soil based geopolymer with fly ash, soil based geopolymer with GGBS, and soil based geopolymer with fly ash and GGBS on compression strength at (**a**) 1 day and (**b**) 7 days curing.

The unconfined compression test was used to evaluate the optimum mix design for soil stabilized with geopolymer. Figure 4a,b presents the unconfined compression results for different solid to liquid (S/L) ratios for soil stabilized with a geopolymer process for 1 day and 7 days of curing. It can be seen that the unconfined compressive strength showed an increasing trend of the S/L ratio until the S/L ratio of 1.5, then the result of compressive strength gradually dropped with the increase in the S/L ratio. The highest strength obtained for soil based geopolymer, soil based geopolymer with fly ash, soil based geopolymer with GGBS, and soil based geopolymer with fly ash and GGBS were recorded at an S/L ratio of 1.5 with the value of 0.57, 1.412, 3.15, and 2.27 MPa respectively.

The strength for all samples of composites soils starts to decrease when the S/L ratio reached 2.0. At an S/L ratio of 2.0, the soil based geopolymers sample was less workable, and homogenous mixtures could not be obtained due to the low amount of alkaline activator. In addition, the amount of liquid was very low compared to solid at this ratio. These cause difficulties in mixing the component, thus affecting the workability of geopolymer samples. Therefore, the compaction and release of samples during the molding process will also be problematic. The samples prepared were porous, thus leading to the reduction in strength. This explains the decrease of strength value obtained for all soil based geopolymer samples at an S/L ratio of more than 1.5, indicating that the S/L ratio gives a significant effect on the compressive strength and workability of the soil sample. This finding is in line with a previous study conducted by Zaliha et al. [26], which found that the optimum ratio of S/L produced soil based geopolymer samples with optimum strength was at an S/L ratio of 1.5. As the S/L ratio increased, the solid content of the mixture increased, while the decrease in liquid content results in decreased workability. A previous study also supported this finding, which suggested that the higher the S/L ratio used, the lower sample workability, which causes a reduction in the homogeneity of the soil based geopolymer sample [31].

On the other hand, the increment of unconfined compressive strength for all soil based geopolymer samples cured at 1 compared to 7 days curing for the solid:liquid (S/L) ratio 1.0:3.0 is more than 50%. This is caused by geopolymeric reactions that occur when GGBS, fly ash particles, and alkali activator (sodium hydroxide (NaOH) and sodium silicate (Na_2_SiO_3_) were added to the clay soil. The primary mechanism involves fly ash and ground granulated blast slag (GGBS) subjected to alkaline activation, which has both geopolymeric and CASH gel-forming concurrently. The strength development of alkali-activated fly ash and GGBS increases with a more significant amount of calcium in fly ash and slag material as a result of more considerable calcium dissolution and precipitation of CASH gel [50,51]. The formation of CASH gel in the alkali-activated material due to the increasing amount of calcium content led to the enhanced strength, as shown in Figure 4.

According to ASTM D4609 [19], the increase in unconfined compression test value of more than 345 kPa for soil stabilization is considered adequate. Based on the results of compression strength, the samples of soil based geopolymer with GGBS and fly ash showed an increment of more than 345 kPa in the strength values. According to the Design Guideline for Alternative Pavement Structures (Low Volume Roads) of Malaysia Public Work Department (PWD) [21], the stabilized subgrade must have a minimum unconfined compression test of 0.8 MPa. The UCT results indicated that the soil based geopolymer with GGBS and fly ash samples could be used as the road subgrade since the values achieved were more than 0.80 MPa. It can be concluded that the geopolymerization method has proven effective in improving the strength of the soils.

### 3.3. Atterberg Limit Test

**Figure 5:**
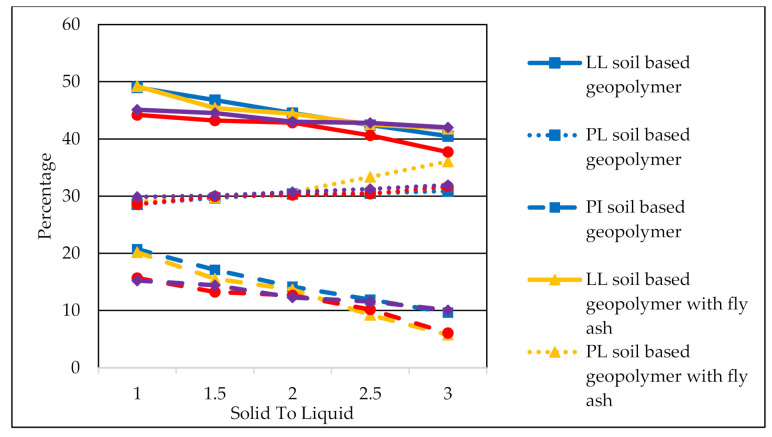
Limits of soil, soil based geopolymer, soil based geopolymer with fly ash, soil based geopolymer with GGBS, and soil based geopolymer with fly ash and GGBS with different solid to liquid (S/L) ratio (LL: liquid limit; PL: plastic limit; PI: plasticity index).

The Atterberg limits of all soil based geopolymer samples with different solid to liquid ratios are presented in Figure 5. The trend of liquid limit, plasticity index, and plastic limit of the soil based geopolymer, soil based geopolymer with fly ash, soil based geopolymer with GGBS, and soil based geopolymer with fly ash and GGBS samples could be observed as the solid to liquid (S/L) ratio increased. All geopolymer soils showed a reduction of plasticity index and liquid limit and an increment of the plastic limit with the increment of the S/L ratio. The plasticity index and liquid limit values were reduced with the increase of the S/L ratio at 1.0 S/L ratio and a decrease at 3.0 S/L ratio. Furthermore, the plastic limit values increased with an improvement in the S/L ratio from 1.0 to 3.0 S/L ratio.

Increasing S/L resulted in a decrease in liquid limit and plasticity index (indirectly proportional) and an increase in plastic limit in all forms of geopolymer soil (directly proportional). The reduction of alkaline activator content resulted in these patterns. Sarathi Parhi et al. [52] reported the same findings on the increment of the S/L ratio, leading to decreased liquid limit and plasticity index and an improvement in the plastic limit. Another study done by Thomas Ansu et al. [22] reported that alkali-activated GGBS aims to raise the plastic limit while lowering the plasticity index and liquid limit as the dosage of alkali activator GGBS is increased. In this study, soil, GGBS, and fly ash act as the aluminosilicate materials, and an alkali activator was used as a stabilizer.

The reduction of liquid limit and increment of plastic limits were due to the cation exchange, the flocculation, and the agglomeration process of clay soil particles. The introduction of a binder (GGBS and fly ash) into the soil releases calcium (Ca^2+^) ions in exchange for metal (Na^+^) ions which are incorporated in clay soil particles. The exchange process of these ions is referred to as cation exchange which produces many physical changes in the soil [43]. The first change that occurs is a change in strength and texture in the soil, such as the transition from the high plastic clay to a friable soil which is characterized by a low plasticity index. Next, the flocculation/agglomeration process in the soil is a process where soil particles that are scattered in a solution stick together, forming flakes or cluster clumps of a larger size. Agglomeration and flocculation occur after mixing, which involves restructuring the negatively charged clay soil particles enveloped by the positively charged cation shells [53].

According to ASTM D 2487 [35] and ASTM D 3282 [54], the stabilized subgrade must have a maximum plasticity index of 20% and a minimum plasticity index of 11%. Based on the results of the Atterberg limits, soil based geopolymer samples with GGBS and fly ash could be used as the road subgrade. Thus, this indicates that the geopolymerization method has proven effective in reducing the plasticity index of the clay soil.

### 3.4. Phase Analysis

**Figure 6:**
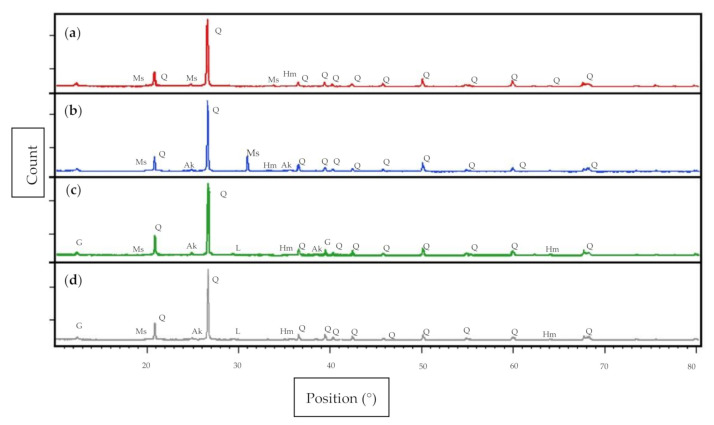
XRD patterns of (**a**) soil based geopolymer, (**b**) soil based geopolymer with fly ash, (**c**) soil based geopolymer with GGBS, and (**d**) soil based geopolymer with fly ash and GGBS at optimum S/L ratio. Q: quartz (ICCD reference: 01-089-1961); Hm: hematite (ICDD reference: 00-024-0072); Ms: muscovite (ICCD reference: 00-001-1098); Ak: akermanite (ICDD reference: 00-035-0592); G: gypsum (ICDD reference: 00-033-0311); L: Larnite (ICDD reference: 00-031-0299).

Figure 6 illustrates the XRD pattern of the soil based geopolymer, soil based geopolymer with fly ash, soil based geopolymer with GGBS, and soil based geopolymer with fly ash and GGBS at optimum S/L ratio. The XRD pattern of soil based geopolymer with the best design showed that it contains quartz (SiO_2_) as a major mineral, and some minor mineral of hematite (Fe_2_O_3_) was detected at 2θ values of 26.66° and 35.62°. The XRD patterns of soil geopolymer produced a new peak of muscovite (Al_2_K_2_O_6_Si) that was narrow at 2θ values of 33.79°. For soil based geopolymer with fly ash, the presence of quartz (SiO_2_) peaks was detected as a major mineral that can be seen in spectra of soil based geopolymer with fly ash samples at 2θ values of 26.63° and some minor minerals of hematite (Fe_2_O_3_), muscovite (Al_2_K_2_O_6_Si) and akermanite (2CaO·MgO·2SiO_2_) were detected at 2θ values of 33.19°, 20.84°, and 24.85°, respectively. The presence of a new mineral of muscovite (Al_2_K_2_O_6_Si) peaks was detected at a 2θ value of 30.96°.

The presence of quartz (SiO_2_) peaks in the XRD pattern of soil based geopolymer with GGBS samples can be seen in spectra of geopolymer soil with GGBS samples at 2θ values of 26.65°. Some minor minerals, namely hematite (Fe_2_O_3_), muscovite (Al_2_K_2_O_6_Si), gypsum (CaSO_4_·2H_2_O), and akermanite (2CaO·MgO·2SiO_2_), were detected at 2θ values of 33.15°, 19.88°, 12.39°, and 24.90°, respectively. The XRD patterns of soil based geopolymer with GGBS produced a new peak of larnite as the major mineral, and the larnite (Ca_2_SiO_4_) peak was detected at 2θ = 29.44°. For soil based geopolymer with fly ash and GGBS presence of the quartz (SiO_2_), peaks were detected as the major mineral can be seen in the soil based geopolymer spectra with fly ash and GGBS samples at 2θ value of 26.68°. The XRD patterns of soil based geopolymer with GGBS and fly ash indicate the occurrence of peaks of muscovite (Al_2_K_2_O_6_Si), hematite (Fe_2_O_3_), gypsum (CaSO_4_·2H_2_O), akermanite (2CaO·MgO·2SiO_2_), and larnite (Ca_2_SiO_4_) that were narrow at 2θ values of 20.38°, 33.22°, 12.44°, 24.92° and 29.44°, respectively.

Kaolin was found to be totally reactive in geopolymerization process as the peak was absent in Figure 3. The presence of the (Al_2_K_2_O_6_Si) is due to the original source of soil and alkali activator driven by high alumina and silica. This result is supported by the high percentage of SiO_2_ (73.30%) and Al_2_O_3_ (17.00%). The presence of muscovite indicates that the initial phase of kaolin was dissolved in the alkali solution. The existence of muscovite (Al_2_K_2_O_6_Si) minerals has been proven to be capable of helping to control the entry of water into the soil so that the soil does not expand when exposed to water [55].

The existence of akermanite (2CaO·MgO·2SiO_2_), muscovite (Al_2_K_2_O_6_Si), and hematite (Fe_2_O_3_) peaks were consistent with the chemical compound analysis of fly ash tabulated in Table 3. Akermanite (2CaO·MgO·2SiO_2_) is present to control the increase in soil volume, which serves to fill the cavity of soil accurately and improve the mechanical strength of the soil [14].

On the other hand, gypsum (CaSO_4_·2H_2_O), akermanite (2CaO·MgO·2SiO_2_), and larnite (Ca_2_SiO_4_) consist of calcium (Ca). The existence of larnite was due to the crystalline hydration reaction of calcium oxide (CaO), silica (SiO), and aluminum oxide (AlO) released from the geopolymer matrix and the reaction of residual materials where the materials were found in the GGBS based on chemical element analysis of GGBS as presented in Table 3. The presence of gypsum (CaSO_4_·2H_2_O), akermanite (2CaO·MgO·2SiO_2_), and larnite (Ca_2_SiO_4_) improve the strength and reduce the plastic limit and liquid limit of the soil. This finding is supported by Shamshad et al. [15], which proved that the addition of GGBS led to the reduction of plasticity index and liquid limit (indirectly proportional) and improvement of plastic limit (directly proportional).

### 3.5. Morphology Analysis

**Figure 7:**
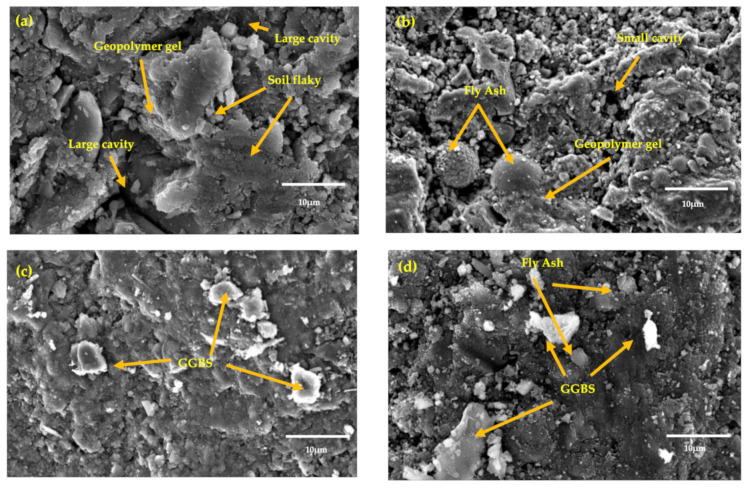
SEM image of (**a**) soil based geopolymer, (**b**) soil based geopolymer with fly ash, (**c**) soil based geopolymer with GGBS, and (**d**) soil based geopolymer with fly ash and GGBS at optimum S/L ratio.

The microstructure of soil based geopolymer, soil based geopolymer with fly ash, soil based geopolymer with GGBS, and soil based geopolymer with fly ash and GGBS at their optimum S/L ratio are shown in Figure 7. Soil based geopolymer samples showed a non-homogeneous geopolymer matrix due to the existence of unreacted soil particles. Soil particle and fly ash particle in soil based geopolymer with fly ash reacted with geopolymer gel. However, the soil and fly ash particles can still be seen. Meanwhile, for soil based geopolymer with GGBS, the best geopolymerization effect was shown where less unreacted particles could be seen. The soil and GGBS particles were fully reacted, leading to a more homogeneous intervening geopolymer matrix, dense and relatively smooth. Lastly, particles in soil based geopolymer with GGBS and fly ash can also be seen to react with the alkaline activator fully. However, unreacted particles can still be observed in non-homogeneous geopolymer.

Soil based geopolymer samples showed a non-homogeneous geopolymer matrix due to the existence of a less dense matrix inside the geopolymer that also leads to low compressive strength. The occurrence of non-homogeneous geopolymer matrix causes the slow reaction between the soil and alkali activator solution and incomplete geopolymerization process due to the curing process performed at room temperature. This finding is in line with Zaliha et al. [26], where the non-homogeneous geopolymer matrix causes the slow reaction between soil and alkali solution and incomplete geopolymerization due to the low temperature of curing.

The soil and fly ash particles were almost entirely covered by the geopolymer matrix. However, soil and fly ash particles and small voids can still be seen. Additionally, spongy particles were also seen covering the spherical particles. Geopolymer gel that accrued in the sample showed geopolymerization reaction in soil and fly ash particle [15]. Non-homogeneous geopolymer and small voids contribute to the reduction in geopolymer strength [26]. However, the presence of fly ash in soil based geopolymer can produce better strength when compared with soil based geopolymer without fly ash.

Soil based geopolymer with GGBS was more homogeneous intervening geopolymer matrix, dense and relatively smooth. The soil and GGBS particles were completely geopolymerized, where the particles are dense, fairly smooth, and surrounded by the geopolymer, hence producing higher strength. The addition of GGBS to soil based geopolymer exhibited the highest strength compared to other geopolymer soil samples. This finding supports the previous study by Phummiphan [14].

The presence of GGBS and fly ash particles filled the cavities between soil particles resulting in the stable and compact structure of clay soil, thus increasing the compression strength. The reduction of GGBS percentage leads to the decrease in the strength compared to soil based geopolymer with GGBS sample due to the contribution of calcium (Ca) in GGBS content that plays a major factor in improving the strength of the geopolymer sample. This finding is similar to those by Shamshad et al. [15], where reduction of GGBS percentage in soil stabilization led to the decrease in soil strength. Ca^2+^ cations sourced from GGBS complement the geopolymer system. The chemical nature of the calcium approved the Ca^2+^ cations to build a strong ionic bond with Si^4+^ through oxygen atoms which are previously connected. At the same time, the extra amount of Ca^2+^ interacted with OH from the water component and generated calcium hydroxide, Ca(OH)_2_. The Ca(OH)_2_ in the system extends the covalent bonding in the geopolymer matrix as a result of the formation of CaCO_3_ bonded with three oxygen atoms by covalent bonds. The corresponding formation of hydration products could contribute to high compressive geopolymer properties [51].

### 3.6. Functional Group Analysis

**Figure 8:**
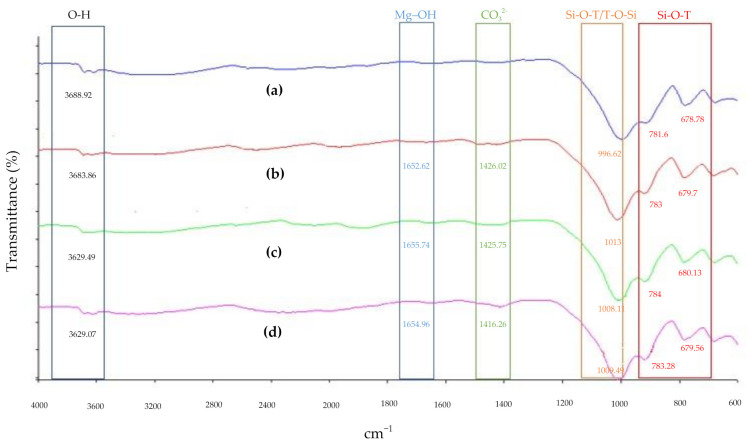
FTIR analysis of of (**a**) soil based geopolymer, (**b**) soil based geopolymer with fly ash, (**c**) soil based geopolymer with GGBS, and (**d**) soil based geopolymer with fly ash and GGBS at optimum S/L ratio.

The structural bonding of the control soil based geopolymer, soil based geopolymer with fly ash, soil based geopolymer with GGBS, and soil based geopolymer with fly ash and GGBS at optimum S/L ratio were obtained using FTIR, as shown in Figure 8. The O–H symmetric was monitored at 3629.07 to 3688.92 cm^−1^, which represents the water molecules. The absorption bands at 1652.62 to 1655.74 cm^−1^ indicate the Mg-OH band of magnesium mineral. Another absorption band at 1416.26 to 1426.02 cm^−1^ had been reported due to the stretching CO_3_^2−^ ions, revealing traces of carbonates. The primary structural bands are located at the peaks, which are 900 and 1009 cm^−1,^ showing Si-O-T asymmetric stretching.

The tightening of peaks was checked between the spectra of soil based geopolymer, soil based geopolymer with fly ash, soil based geopolymer with GGBS, and soil based geopolymer with fly ash and GGBS. A shift in peaks decreased O–H symmetric wavenumbers, from 3688.92 to 3629.07 cm^−1^ of all geopolymer soil samples. This indicates the removal of free water in the geopolymer sample at room temperature. A similar result was reported by Tigue et al. [23,27], where the addition of fly ash and GGBS to geopolymer soil can cause a shift in peaks to decrease the wavenumbers of O–H symmetric. The decreasing wavenumber of O–H symmetric represents the contribution of water molecules in the sample. In other words, this indicates that the addition of GGBS and fly ash can absorb water to control the water content in the sample of soil, thus improving the mechanical strength of the soil [22,23,24,25].

The band CO_3_^2−^ stretching was observed at 1416.26 to 1426.02 cm^−1^, which represents larnite as a reaction between excess atmospheric carbon dioxide with calcium oxide (CaO), silica (SiO), and aluminum oxide (AlO) released from the geopolymer matrix [51,56,57]. The band at around 1652.62 to 1655.74 cm^−1^ of the Mg–OH bond highlights the presence of magnesium mineral from akermanite (2CaO·MgO·2SiO_2_). This invention is in line with the previous study conducted by Latifi et al. [58], which reported a new absorption band at 1619 cm^−1^ indicating that the Mg-OH bond of magnesium mineral was evident. Besides, Arioz et al. [59] reported that the band at around 1644 cm^−1^ could also be assigned to the bending vibrations of bond water molecules mainly because the band at about 3593 to 3450 cm^−1^ related to the stretching vibrations of bound water molecules was found.

Peaks within the primary band for soil based geopolymer, soil based geopolymer with fly ash, soil based geopolymer with GGBS, and soil based geopolymer with fly ash and GGBS were obviously more pointed than the geopolymer soil sample without additional materials. This is significant to the change in the matrix structure of the geopolymer bonds affected by the addition of GGBS and fly ash [45,46]. The altering peaks which shift the wavenumbers of Si–O–T asymmetric stretching and Si–O–T bending vibration, from 996.62 to 1013 cm^−1^ and 678.78 to 784 cm^−1^ of geopolymer soil samples indicated that the stabilization/solidification process of the geopolymer is a chemical reaction and the geopolymerization is active at room temperature [60]. This finding is in line with a previous study conducted by Sedira et al. [60] that reported a shift in peaks to increase the wavenumbers of Si–O–T asymmetric stretching and Si–O–T bending vibration, clearly indicated that the alkali-activated could cause an increase in the compression strength. This finding proves that the geopolymerization method effectively increases the mechanical and physical properties of the soil based on the increase in compressive strength of the geopolymer soil with GGBS and fly ash.

## 4. Conclusions

Soil stabilization using ground granulated blast slag (GGBS) and fly ash through geopolymerization process to increase the compression strength and control the Atterberg limit of clay soil has been investigated. Based on the results obtained after the addition of GGBS and fly ash through the geopolymerization process, it can be concluded as follows:

- The effect of the S/L ratio and the optimum S/L ratio was obtained at the ratio of 1.5 for all geopolymer soil samples. This ratio was found to have produced workability for stabilized geopolymer soil in the mixing process, thus increased the unconfined compression test of the geopolymer soil.
- Based on the compression test results, the geopolymer soil with GGBS and fly ash could be used as the road subgrade since the values achieved were more than 0.8 MPa. This indicates that the soil stabilization using fly ash and GGBS based geopolymer has proven effective in increasing the strength of the soil according to the ASTM D4609 standard and Design Guideline for Alternative Pavement Structures (Low Volume Roads) of Malaysia Public Work Department (PWD).
- The XRD diffractogram indicates the presence of gypsum (CaSO_4_·2H_2_O), akermanite (2CaO·MgO·2SiO_2_), and larnite (Ca_2_SiO_4_) of geopolymer soil with fly ash and GGBS, which had improved the strength and reduced the plastic limit and liquid limit of the clay soil.
- The morphology showed that the soil and GGBS particles were fully reacted; more homogeneous intervening geopolymer matrix, dense and fairly smooth. The existence of additional GGBS and fly ash particles filled the void between clay soil particles resulting in the stable and compact structure of clay soil, thus increasing the compression strength.

Further investigation on soil stabilization is needed so that it can be applied to road pavement construction, especially on subgrade layers. However, many factors affect the functional performance of geopolymer soil stabilization. It can be difficult to reach clear conclusions due to the variety of raw materials. Further improvements in the aspect of performance and durability, such as precursor reactivity index, setting time, drying shrinkage, and alkali reactions, are required. In addition, other methods of investigating the effects of using different molarity of sodium hydroxide (NaOH) and sodium silicate/sodium hydroxide (Na_2_SiO_3_/NaOH) ratio on soil properties and further studies on the effects of various curing temperatures on geopolymer soil are also needed.

In addition, the cost of geopolymer raw materials is high due to strict legal limits on resource extraction in many countries and regions. Furthermore, standardization is a problem. Until entering the market, newly developed soil stabilized with geopolymer must meet the requirements of national and foreign authorities.

## Figures and Tables

**Table 1:** Mix design.

Sample	Percentage Blended Mix Proportion	NaOH Concentration (M)	S/L Ratio	Na_2_SiO_3_/NaOH Ratio
Soil based geopolymer-1	100% Soil	10	1.0	2.0
Soil based geopolymer-2	100% Soil	10	1.5	2.0
Soil based geopolymer-3	100% Soil	10	2.0	2.0
Soil based geopolymer-4	100% Soil	10	2.5	2.0
Soil based geopolymer-5	100% Soil	10	3.0	2.0
Soil based geopolymer with fly ash-1	80% soil and 20% Fly ash	10	1.0	2.0
Soil based geopolymer with fly ash-2	80% soil and 20% Fly ash	10	1.5	2.0
Soil based geopolymer with fly ash-3	80% soil and 20% Fly ash	10	2.0	2.0
Soil based geopolymer with fly ash-4	80% soil and 20% Fly ash	10	2.5	2.0
Soil based geopolymer with fly ash-5	80% soil and 20% Fly ash	10	3.0	2.0
Soil based geopolymer with GGBS-1	80% Soil and 20% GGBS	10	1.0	2.0
Soil based geopolymer with GGBS-2	80% Soil and 20% GGBS	10	1.5	2.0
Soil based geopolymer with GGBS-3	80% Soil and 20% GGBS	10	2.0	2.0
Soil based geopolymer with GGBS-4	80% Soil and 20% GGBS	10	2.5	2.0
Soil based geopolymer with GGBS-5	80% Soil and 20% GGBS	10	3.0	2.0
Soil based geopolymer with GGBS and fly ash-1	80% Soil 10% GGBS and 10% Fly ash	10	1.0	2.0
Soil based geopolymer with GGBS and fly ash-2	80% Soil 10% GGBS and 10% Fly ash	10	1.5	2.0
Soil based geopolymer with GGBS and fly ash-3	80% Soil 10% GGBS and 10% Fly ash	10	2.0	2.0
Soil based geopolymer with GGBS and fly ash-4	80% Soil 10% GGBS and 10% Fly ash	10	2.5	2.0
Soil based geopolymer with GGBS and fly ash-5	80% Soil 10% Fly ash and 10% GGBS	10	3.0	2.0

**Table 2:** Properties of clay soil, fly ash and ground granulated blast slag.

Properties	Unit	Soil	Fly Ash	GGBS
i. Particle Analysis:				
ii. Fine Particle	%	52.00	94.00	96.00
iii. Course Particle	%	48.00	6.00	4.00
Specific Gravity	-	2.686	-	
i. Atterberg Limit:	-	-	-	-
ii. Liquid Limit	%	51.20	23.40	40.73
iii. Plastic Limit	%	28.48	Non-plastic	Non plastic
iv. Index Plasticity	%	22.72	Non-plastic	Non plastic
USCS Classification	-	(CH)	-	-
		Inorganic clay and high plasticity	-	-
Optimum Water Content	%	15	-	-
Optimum Dry Density	gram/cm^3^	1.66	-	-

**Table 3:** Percentage of chemical elements of soil, fly ash and GGBS.

Element	Clay Soil (%)	Fly Ash (%)	GGBS (%)
SiO_2_	73.30	30.70	30.40
Al_2_O_3_	17.00	13.30	10.50
Fe_2_O_3_	6.15	23.92	-
CaO	-	22.40	50.37
MgO	-	3.6	3.2
Others	3.55	6.08	5.53

## Data Availability

The data underlying this article will be shared on reasonable request from the corresponding author.
